# Factor Analysis Demonstrates a Common Schizoidal Phenotype within Autistic and Schizotypal Tendency: Implications for Neuroscientific Studies

**DOI:** 10.3389/fpsyt.2014.00117

**Published:** 2014-08-27

**Authors:** Talitha C. Ford, David P. Crewther

**Affiliations:** ^1^Centre for Human Psychopharmacology, Faculty of Health, Arts and Design, School of Health Sciences, Swinburne University of Technology, Melbourne, VIC, Australia

**Keywords:** autistic traits, schizotypal personality traits, schizoid personality disorder, factors analysis, autism, schizophrenia

## Abstract

Behavioral and cognitive dysfunction, particularly social and communication impairments, are shared between autism and schizophrenia spectrum disorders, while evidence for a diametric autism-positive schizophrenia symptom profile is inconsistent. We investigated the shared phenotype at a personality trait level, particularly its resemblance to schizoid personality disorder, as well as differential aspects of the autism–schizophrenia model. Items of the autism spectrum quotient (AQ) and schizotypal personality questionnaire (SPQ) were pseudo-randomly combined, and were completed by 449 (162 male, 287 female) non-clinical participants aged 18–40. A factor analysis revealed three factors; the first represented a shared social disorganization phenotype, the second reflected perceptual oddities specific to schizotypy while the third reflected social rigidity specific to autism. The AQ and SPQ were strongly correlated with Factor 1 (AQ: r = 0.75, p < 0.001; SPQ: r = 0.96, p < 0.001), SPQ score was correlated with Factor 2 (r = 0.51, p < 0.001), particularly in cognitive–perceptual features (r = 0.66, p < 0.001), and AQ score was strongly correlated with Factor 3 (r = 0.76, p < 0.001). Furthermore, there was no relationship between Factor 1 and Factor 2. Thus, there is robust evidence for a shared social disorganization phenotype in autistic and schizotypal tendency, which reflects the schizoid phenotype. Discriminating and independent dimensions of schizotypal and autistic tendency exist in Factors 2 and 3, respectively. Current diagnostic protocols could result in different diagnoses depending on the instrument used, suggesting the need for neuromarkers that objectively differentiate autistic and schizotypal traits and resolve the question of commonality versus co-morbidity.

## Introduction

The phenotypic tangle of autism and schizophrenia spectrum symptomology has been hotly debated since Bleuler defined “autism” in 1911 as an exclusive psychiatric disorder (1, 2). Despite their obvious clinical differences in symptom onset and presentation, interpersonal and cognitive deficits, and disorganization are fundamental to both disorders (1, 3–9), yielding a potential confusion in diagnosis.

Autism and schizophrenia (the terms “autism” and “schizophrenia” refer to the full spectra of the respective disorders) are neurodevelopmental disorders with pervasive social impairments such as flattened facial and speech affect, reduced gesturing, eye contact and language, concrete and obsessional thinking, and unusual body movement (10). King and Lord (11) pointed to symptom similarity between schizotypal personality and autism in terms of unusual preoccupations, unusual perceptual experiences, odd thinking and speech, constricted affect, social anxiety, lack of close friends, and odd or eccentric speech and behavior. A clinical diagnosis of autism spectrum disorder (ASD) specifies early childhood presentation of social and communication dysfunction, in conjunction with restricted and repetitive behaviors (10). Schizophrenia is typically qualified with the onset of a psychotic episode, marked by hallucinations, delusions, disorganization, and/or catatonic behavior for up to 1 month, in late adolescence or early adulthood. Schizophrenia diagnoses can be established only if the psychosis is accompanied by enduring affective and interpersonal dysfunction, and disorganization in speech and behaviors (10).

The newly released Diagnostic and Statistical Manual of Psychiatric Disorders version five (DSM-5) (10) is naturally controversial due to its central role in clinical diagnosis, and the revision of symptom discrimination, differentiation, and co-morbidity. The new edition saw the removal of paranoid and schizoid personality disorder (PD), which may be detrimental to diagnostic specificity. Schizoid PD, as defined in DSM-IV-TR (12), describes pervasive social dysfunction and negative symptoms that are central to schizophrenia (including schizotypal PD). Such symptoms are also seen in autism. Core features of schizoid PD include: lack of interest in, and active avoidance of social situations both in occupation and daily life, restricted affect, odd communication, relationship detachment, and poor empathy. Mental rigidity and single-minded pursuit of interests are also characteristic (12–14). These features can indirectly lead to positive-like symptoms such as fantasies, mania (13), and paranoid ideation (11). Schizoid PD, thought to be a milder form of schizotypal PD, has the potential to progress into more enduring schizophrenia spectrum disorders, with a genetic association to schizophrenia (14, 15). In fact, in a study of 32 children diagnosed with schizoid PD, 24 met the criteria for schizotypal PD, and two developed schizophrenia (14). Exclusion of schizoid PD was based on a lack of empirical evidence for the disorder. Schizoid PD was reported to have only 1% pathological prevalence (13), compared to 3% prevalence of schizotypal PD (12).

DSM-5, in similar vein, aligns Asperger’s disorder with ASD resulting in the abolishment of key differential diagnostic criteria. Removing language delay and early onset symptom presentation criteria for ASD consequently reduces the discriminatory quality of diagnosis. Wolff et al. (14) suggest distinguishing between schizotypal PD, schizoid PD, and Asperger’s disorder is not warranted. Thus, the relaxed exclusion criterion for ASD, and removal of schizoid PD, impacts the diagnosis, prognosis, and therapeutic techniques for autism and schizophrenia spectrum disorders. More accurate assessment of the schizoid phenotype may indeed reduce confusion between the apparent comorbid social dysfunction in the autism and schizophrenia spectra (13).

Social–cognitive dysfunction is evident in both autism (7, 16–19) and schizophrenia (7, 16–22). Social cognition is defined as the cognitive aspects of the social experience; including perceptions, processing, and interpreting social information (23) from basic facial affect recognition to theory of mind (22). Social anhedonia – social isolation and disinterest, is a prodromal, as well as an active and residual feature of schizophrenia (24, 25). Social anhedonia has also been found to predict severity in autism (26). However, it has been argued that social deficits in autism represent social anxiety and social skills, while negative schizotypy relates to social anhedonia and depression (27). The similarity between the two disorders in terms of social cognition and interpersonal deficit may lead to confusion in symptom interpretation, and consequently result in misdiagnosis (9, 22).

Due to the spectrum nature of both disorders, symptoms in the general population grade from clinical pathology to personality traits (1). Self-report measures such as the autism spectrum quotient (AQ) (28) and Schizotypal Personality Questionnaire (SPQ) (29) reliably identify autistic and schizotypal traits, respectively among clinical (3, 4, 6, 30–32) and non-clinical populations (5, 33–35). The AQ contains five subscales that reflect the DSM-IV criteria for autism: social skills, attention to detail, attention switching, communication, and imagination (36). The SPQ provides a measure of schizotypal tendency in accordance with the nine DSM-III-R criteria for schizotypal PD (37). Three superordinate dimensions encapsulate these nine subscales: Ideas of reference, odd beliefs, unusual perceptual experiences, and suspiciousness (cognitive–perceptual/positive); excessive social anxiety, no close friends and constricted affect (interpersonal/negative); odd behavior and odd speech (disorganized) (29, 38). A brief version of the SPQ (SPQ-B) was introduced in 1995 by Raine and Benishay (39) and then revised in 2010 by Cohen et al. (SPQ-BR) (40). Cohen et al. (40) reduced the SPQ to 32-items within seven subscales, uniting Ideas of Reference and Suspiciousness, and No Close Friends and Constricted Affect (40). Despite the obvious benefits of creating a briefer scale, the full-scale SPQ provides a comprehensive measure of schizotypy based on schizotypal PD, unlike the brief versions revised based on non-clinical student samples. Furthermore, in reducing the number of response opportunities, valuable information about the diversity of the schizotypal phenotype may well be missed. The SPQ-BR has only three or four items representing each of the nine criteria for schizotypal PD (40), compared with the seven to nine questions representing the same criteria in the full-scale SPQ (29). This relative paucity of assessment may result in noisier data, likely affecting diagnostic predictive power.

Autistic traits are particularly similar to disorganized (3–6) and interpersonal features of the SPQ (3, 5, 33, 34). Furthermore, Schizotypal tendency, quantified by the SPQ, is significantly higher in autism (3, 6) and Asperger’s disorder (4, 5) than in controls, while AQ-measured autistic tendency is higher in schizophrenia (3, 31, 32). The common social–interpersonal dysfunction and communication–disorganization in schizotypal PD and Asperger’s disorder may reflect true comorbid symptoms. Alternatively, the apparent co-morbidity could result from a lack of differentiation between distinct symptoms by measurement tools (5).

The diametric model argues that positive or cognitive–perceptual features are opposed to the social aspects of autism (3, 5, 33–35). The AQ’s imagination subscale quantifies a rigidity of thought and convergent thinking, which is in contrast with the fluidity of thought characterizing schizophrenia (5, 41). Crespi and Badcock (2) suggested that social–cognitive dysfunction in autism is diametric to that in schizophrenia. Specifically, that social–cognitive dysfunction is under-developed in autism and over-developed in schizotypy (leading to hyper-developed theory of mind). Nevertheless, positive schizotypal features remain stronger in autism than controls (6, 35), and paranoid thinking in autism and schizophrenia may be a subsequent consequence of social–communication misperceptions (11). Furthermore, SPQ unusual perceptual experiences and Odd behavior’s mimic AQ predictors of abnormal sensory responses and restrictive/repetitive behaviors, respectively (4). Altogether, the core social dysfunction, with evidence of broad trait similarities provides support for a shared schizoid phenotype in autism and schizophrenia spectrum disorders.

Dinsdale et al. (35) supported a shared social and communication dysfunction in autistic and schizotypal tendency, as well as supporting the diametric model of positive schizotypy and autism. The authors ran a principal component analysis (PCA) of combined AQ and SPQ-BR subscales revealing two components. The first component reflected social–communication disinterest, impairment, and abnormalities with predominant contributions from the AQ subscales social skills and communication, and SPQ-BR subscales constricted affect, social anxiety, odd behavior, and ideas of reference. The second component reflected a pattern of diametric social autism and positive schizotypy. Substantial contributions from SPQ-BR subscales Odd beliefs and unusual perceptions loaded positively and AQ subscales of social skills and imagination loaded negatively create an autism-positive schizotypy axis (35). The authors also carried out their own PCA analysis of Wakabayashi et al.’s (34) full-scale SPQ and AQ data (35). The resulting two-component solution supported their own PCA results, however, differences in subscale contribution to the components suggests that the full-scale SPQ provides a more robust division of subscales than does the brief form. Specifically, attention switching was exclusive to the first component, odd behavior contributed equally to both components, and ideas of reference contributed more substantially to the second component (35). Put simply, in Wakabayashi et al.’s dataset, the first component appears to better represent a social behavioral dysfunction, while the second gives a stronger representation of cognitive–perceptual and disorganized subscales. Overall, the first component from both datasets supports a co-morbidity of traits within the broader social dysfunction phenotype in autistic and schizotypal tendencies, particularly those specific to Asperger’s disorder and schizoid PD (35).

It is noteworthy that in these studies, the questionnaires were presented individually on different response scales (AQ: 4-point scale, SPQ-BR: 5-point scale, SPQ: 2-point scale), affecting response specificity and statistical analysis. Furthermore, the total contribution of the components to the total variance in their data was quite low in both datasets, at around 45%. PCA may not be the ideal analysis for this type of data, as it aims to simply reduce a large dataset to a smaller set of components exploring patterns in the data (42). All of the variables variance is included in the PCA, limiting the capacity to identify meaningful underlying constructs, thus, rendering it uninterpretable (43, 44). Factor analysis, on the other hand, can more accurately reveal the underlying constructs as only the variance that is shared among the variables is analyzed (42, 45). Factor analysis is recommended when there is a theoretical basis for a conceptual relationship between the variables (43), thus, this study will adopt factor analysis as the preferred method, and PCA simply to compare with Dinsdale et al. (35).

Co-morbidity between the disorders is seen at a clinical level. Solomon et al. (7) found that 20% of their high risk and first episode schizophrenia participants also met the criteria for autism. Waris et al. (8) identified pervasive developmental disorder (PDD – the diagnostic category in which ASD lies) in 10 of 18 adolescents with schizophrenia, and Rapoport et al. (46) found 20–30% of children with schizophrenia had prodromal and comorbid PDD, and expressive and receptive language deficits. Also, stress-induced behavior in autism can be additionally or misdiagnosed as schizophrenia (47, 48). These studies give evidence of the risk of incorrect behavioral assessment in autism and schizophrenia. Children with Asperger’s were indistinguishable from “loner” (parent rated schizoid personality traits) children on a schizoid scale (49) suggesting potential misclassification of schizoid PD as Asperger’s disorder due to comorbid schizoid trait in “loner” and Asperger’s children. Schizoid PD, until its removal, was differentiated from schizotypal PD in its lacking positive symptom, identical to the distinction of autism from schizotypal PD (12). Misdiagnosis must be avoided in order to eliminate wrongly prescribed psychopharmacological medications, which may have limited success, instead exposing patients to potentially harmful side effects.

The argument for a shared phenotype is further reinforced by genetic and neuroimaging studies, providing an objective link between the disorders. Genome-wide association studies have found genetic overlap in copy number variants between schizophrenia and autism, suggesting similar processes in the development and regulation of synaptic transmission that influence common biological pathways in the two disorders (50). The heritability within and between autism (2, 50, 51) and schizophrenia (2, 10, 12, 50, 52) evidences a common biological foundation between the disorders (46, 50, 53, 54). Furthermore, schizoid traits are more likely in parents of children with autism (55), and parents of children with autism more likely to have a history of a mental disorder, particularly schizophrenia, than control parents (56). Similar social–cognitive neural dysfunction in conjunction with genetic associations further supports the schizoid phenotype as a link between autism and schizophrenia. Altogether, these studies underline adverse implications in the subjective nature of the DSM clinical classification process (50, 57).

Neuroimaging studies directly comparing autism and schizophrenia identify a neural network related to social cognition (18, 58) and other functional and structural similarities (19, 22, 58–62). Gray matter reduction around the STS and limbic-striato-thalamic network is associated with the degree of autistic tendency in schizophrenia and autism (31, 59, 61, 62). Metabolite similarities, such as glutamate, glutamine, gamma-aminobutyric acid (GABA), and *N*-acetylaspartylglutamic acid (NAAG) are related to negative symptoms of schizophrenia (63, 64) and autism (65), and have also been associated with social–cognitive dysfunction in both disorders [see Rossignol for a review (65, 66)]. On the other hand, reduced *N*-acetylaspartyl acid (NAA) has been associated with more severe symptoms in schizophrenia, particularly positive symptoms (64, 67) and reduced social functioning, but not negative symptoms alone (67). Reductions in NAA have also been identified in autism (65, 68), suggesting a common neurotransmitter link between the spectrum disorders and opposing the argument for diametric disorders.

To our knowledge, no previous study has explored the factor structure of a combined, pseudo-randomized version of the original SPQ and AQ (ASQ). Items were presented on a four-point Likert to reduce response bias and yield more reliable participant reports (69). The aim of this study was to extend Dinsdale et al.’s findings via a PCA of the complete ASQ followed by factor analysis in order to identify the underlying constructs (43–45). Furthermore, the study aimed to uncover a more robust phenotypic model for autism and schizophrenia spectrum disorders at a trait level, with particular interest in the schizoid phenotype. It was expected that, as with Dinsdale et al. (35), the ASQ PCA would reveal a factor specific to social AQ (social skills, communication, and attention switching) and Interpersonal SPQ (no close friends, constricted affect, and social anxiety), reflecting schizoid PD. In using only the shared variance in the model, and allowing the resulting factors to correlate, this research explored how cognitive–perceptual subscales (ideas of reference, odd beliefs, unusual perceptual experiences, and suspiciousness) contributed to the model. It was expected that the disorganization subscales would contribute across factors, as these traits are related to both social AQ and interpersonal and cognitive–perceptual dysfunction. In terms of the factor analysis, we expected that a similar model structure would emerge, but that this would provide a more robust model of the underling constructs within autistic and schizotypal traits. Furthermore, we predicted a strong relationship between the interpersonal dimension of the SPQ and AQ social subscales: social skills, attention switching, and communication.

## Materials and Methods

### Participants

Participants were sourced through social media and advertisements targeting the general population. A total of 449 adults aged between 18 and 40 years, 162 males (mean = 24.20, SD = 4.92) and 287 female (mean = 23.08, SD = 5.01), volunteered for the study, accessing and completing a combined questionnaire online. On average males were older than females [one-way ANOVA, *F*(1,448) = 5.22, *p* < 0.05]. The Swinburne University Human Research Ethics Committee approved the collection of participant data; informed consent was obtained from each participant prior to completing the questionnaire.

### Materials

Autistic tendency was measured with Baron-Cohen et al.’s (28) AQ comprising 50 items within five subscales: social skills, attention switching, attention to detail, communication, and imagination. Schizotypal tendency was quantified using Raine’s (29) 74-item SPQ. The SPQ has nine subscales in accordance with the DSM III-R diagnostic criteria of schizotypal PD, which represent the three core criteria of schizophrenia: cognitive–perceptual (ideas of reference, odd beliefs, unusual perceptual experiences, and suspiciousness), interpersonal (social anxiety, no close friends, and constricted affect), and disorganized (odd behavior and odd speech) (12, 29, 36, 38). Including the full-scale SPQ provided a richer schizotypal trait dataset, while also allowing the extraction of the 32-items that create the SPQ-BR. Subsequently, comparisons against both Dinsdale et al.’s and Wakabayashi et al.’s findings were made (35). Furthermore, we were able to identify any potential confounds of the SPQ-BR, as highlighted previously in relation to the PCA of Wakabayashi et al.’s data (34) conducted by Dinsdale et al. (35).

The original dichotomous “yes/no” response format of the SPQ raises concerns over trait insensitivity and social desirability response bias (70). A Likert scale design has been shown to improve internal reliability and convergence of the SPQ (70) and consequently this study employed a 4-point Likert scale to align with the AQ. Thus, creating a cohesive set of items that was not particularly associated with either questionnaire. The AQ and SPQ items were then combined, pseudo-randomized, and presented online with Opinio (71). Participant responses to combined AQ and SPQ (ASQ) items ranged from 1: “strongly agree,” to 4: “strongly disagree.” In broadening the response options from yes/no (2-point), and removing the “neutral” option in the 5-point scale, the opportunity for respondents to make a conservative response to potentially socially undesirable questions is reduced. There was acceptable internal consistency for SPQ total (α = 0.86) and its superordinate subscales (cognitive–perceptual α = 0.77; interpersonal α = 0.77; disorganized α = 0.69), AQ total (α = 0.66), and ASQ total (α = 0.88).

### Procedure

Participants volunteered to complete the online ASQ through the Opinio website (71). Raw ASQ item scores were converted to zero (0) for an unendorsed response (“strongly disagree” or “disagree”) and one (1) for an endorsed response (“strongly agree” or “agree”). AQ and SPQ items were then extracted from the combined questionnaire to obtain conventional AQ (/50) and SPQ (/74) scores. Participants’ individual subscale and total scores were entered into SPSS Version 20.0 for statistical analysis (72).

### Data analysis

An initial one-way analysis of variance (ANOVA) for gender differences in total AQ and SPQ score was performed. Pearson correlations were obtained within and between AQ and SPQ total and individual subscale scores.

As previously discussed, dimension reduction with PCA is not ideal for data that is interrelated. Therefore, factor analysis was the primary technique in this study. In order to directly compare these data with Dinsdale et al. (35), a PCA including all nine SPQ and five AQ subscales was also conducted. The 32-items of the SPQ-BR were then extracted and a PCA with the seven SPQ-BR and five AQ subscales was conducted (35, 40). Finally, the full-scale ASQ was subjected to a factor analysis with maximum likelihood estimation. Due to the well-reported relationship between AQ and SPQ subscales, there were reasonable theoretical grounds to conduct an oblique (direct oblimin) rotation, taking into account the relationship between the factors (43). The sampling adequacy (Kaiser–Meyer–Olkin measure – KMO) of the data was found to be suitable for each analysis (AQ: KMO = 0.670; SPQ: KMO = 0.887; ASQ: KMO = 0.894; ASBQ: KMO = 0.843). Correlations between the subscales were adequate for factor analysis with Bartlett’s test of sphericity significant for AQ [χ(10) = 387.9, *p* < 0.001], SPQ [χ(36) = 1610.1, *p* < 0.001], ASQ [χ(91) = 2582.3, *p* < 0.001], and ASBQ [χ(66) = 1417.6, *p* < 0.001] (43). Factors/components with Eigenvalues >1.0 were retained as substantial representations of the variation in the model, and the Scree Plot was used as visual support for the retained factors. Subscale contributions to the model were referred to as “factor loadings” and reflected the strength of the relationship between the factor/component and the subscale. Factor loadings below 0.3 were suppressed in order to report only important factor contributions (43). Pearson correlations were obtained between on the resultant factor analysis factors, total AQ, total SPQ, cognitive–perceptual, interpersonal, and disorganized scores.

## Results

The mean AQ and SPQ scores for males and females are shown in Table 1. A one-way ANOVA revealed no significant gender effects on mean AQ score [*F*(1,448) = 0.557, *p* = 0.456], however, there was a significant difference in SPQ score [*F*(1,448) = 4.71, *p* < 0.05]. Participant age did not affect AQ (*r* = 0.031, *p* = 0.507) or SPQ (*r* = −0.006, *p* = 0.904) score.

**Table 1:** **Mean gender difference in AQ and SPQ**.

*N* = 449	*N*	AQ	Min	Max	SPQ	Min	Max
		M (SD)			M (SD)	
Male	162	17.6(6.8)	1	36	24.6(12.6)	2	65
Female	287	17.1(6.6)	1	35	21.8(12.8)	2	61

*AQ, autism spectrum quotient; SPQ, schizotypal personality questionnaire; M, mean; SD, standard deviation*.

### AQ and SPQ subscale correlations

The correlation matrix in Table 2, consisting of total SPQ, total AQ, and all 14 subscales, showed strong correlations between total AQ and total SPQ scores. Each individual subscale was significantly correlated with AQ and SPQ total scores; however, there was only a weak relationship between total AQ and odd beliefs (SPQ), and between total SPQ and imagination (AQ).

**Table 2:** **Correlation matrix for total AQ, total SPQ, and individual subscales**.

	1	2	3	4	5	6	7	8	9	10	11	12	13	14	15
Social skill															
Communication	0.501														
Attention switching	0.38**	0.41**													
Attention to detail	0.40**	0.12*	0.06												
Imagination	0.34**	0.19**	0.11**	0.29**											
AQ total	0.89**	0.67**	0.63**	0.58**	0.55**										
Ideas of ref	0.33**	0.41**	0.42**	0.16**	0.03	0.43**									
Odd beliefs	0.09	0.04	0.07	0.31**	0.03	0.17**	0.40**								
Unusual perceptual exp	0.21**	0.38**	0.34**	0.18**	−0.08	0.33**	0.56**	0.41**							
Suspiciousness	0.38**	0.42**	0.44**	0.21**	0.08	0.48**	0.63**	0.26**	0.47**						
Social anxiety	0.54**	0.50**	0.44**	0.23**	0.11*	0.57**	0.46**	0.13*	0.34**	0.45**					
No close friends	0.58**	0.48**	0.41**	0.23**	0.18*	0.58**	0.46**	0.07*	0.34**	0.51**	0.55**				
Constrict affect	0.52**	0.50**	0.32**	0.29**	0.22**	0.57**	0.38**	0.05	0.30**	0.42**	0.46**	0.62**			
Odd behavior	0.35**	0.52**	0.40**	0.08**	0.07	0.44**	0.47**	0.14*	0.42**	0.42**	0.36**	0.51**	0.45**		
Odd speech	0.31**	0.48**	0.32**	0.25**	0.10*	0.46**	0.52**	0.22**	0.50**	0.51**	0.36**	0.48**	0.50**	0.53**	
Total SPQ	0.54**	0.61**	0.52**	0.30**	0.12*	0.65**	0.79**	0.39**	0.69**	0.76**	0.68**	0.75**	0.70**	0.70**	0.75**

*p < 0.05, **p < 0.001.

Among the individual subscales, the strongest relationships were between social skills (AQ) and communication (AQ) and interpersonal subscales (SPQ): social anxiety, no close friends, and constricted affect. Communication (AQ) also had a robust relationship with the disorganized subscales (SPQ): odd behavior and odd speech. Notably, there were very weak to no relationship detected between imagination (AQ) and all SPQ subscales, and between odd beliefs (SPQ) and all AQ subscales.

### Component and factor structure of combined AQ and SPQ (ASQ)

The PCA of the nine SPQ and five AQ subscales is presented in Table 3 below. The comparison ASQ PCA resulted in a three-component solution. The unique contribution (component loading) of each ASQ subscale to the model was reported in the Pattern Matrix, summarized in Table 3. Scores below 0.3 are not shown.

**Table 3:** **Principal component analysis of combined AQ and SPQ subscales**.

	Component 1	Component 2	Component 3
Communication	0.785		
No close friends	0.782		
Odd behavior	0.740		
Constricted affect	0.697		
Social anxiety	0.691		
Attention switching	0.683		
Suspiciousness	0.628		
Odd speech	0.625		
Ideas of reference	0.593		0.458
Social skill	0.592	0.512	
Attention to detail		0.744	0.446
Imagination		0.724	
Odd beliefs			0.884
Unusual perceptual experience	0.454		0.563
Eigenvalues	5.650	1.574	1.332
Variance explained	40.4%	11.2%	9.5%
Rotation sum of square	5.39	1.80	2.23
Total variance	61.1%		

Subscale: SPQ: 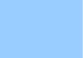 AQ: 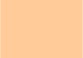

Table 3 illustrates clear overlap of AQ and SPQ subscales, particularly in component 1, which included disorganized (odd behavior and odd speech), interpersonal (no close friends, constricted affect, and social anxiety), cognitive–perceptual (suspiciousness ideas of reference and unusual perceptual experiences) and AQ (communication, social skills, and attention switching) subscales. Component 2 was loaded with imagination, attention to detail, and social skills of the AQ. Finally, component 3 comprised of cognitive–perceptual subscales odd beliefs, unusual perceptual experiences, and ideas of reference, as well as attention to detail from the AQ.

The factor analysis of the ASQ, with an oblimin rotation, resulted in a three-factor solution. The pattern and structure matrix are presented in Table 4. The pattern matrix reports the regression coefficient for each subscale on each factor, that is, the unique contribution that each subscale has to each factor. The structure matrix on the other hand, reports the correlation coefficient between the subscale and factor, thus the factor loading of each subscale takes into account the relationship between factors.

**Table 4:** **Factor analysis pattern and structure matrix of combined AQ and SPQ subscales**.

	Pattern matrix			Structure matrix		
	F1	F2	F3	F1	F2	F3
No close friends	0.738			0.757		0.375
Communication	0.721			0.700		
Odd behavior	0.713			0.679		
Ideas of reference	0.648	0.376		0.701	0.528	
Suspiciousness	0.643			0.691	0.385	
Constricted affect	0.641			0.679		0.413
Odd speech	0.633			0.671	0.332	
Social anxiety	0.623			0.663		0.337
Attention switching	0.605			0.582		
Unusual perceptual experience	0.512	0.438		0.572	0.560	
Odd beliefs		0.721			0.716	
Attention to detail		0.361	0.664		0.330	0.642
Social skill	0.509		0.512	0.620		0.651
Imagination			0.458			0.473
Eigenvalues	5.181	1.091	0.828			
Variance explained	37.0%	7.8%	5.9%			
Rotation sum of square	5.005	1.527	1.605			
Rotation variance explained	30.0%	10.9%	9.8%			
Total variance	50.7%					

*Subscale: SPQ: 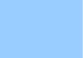 AQ: 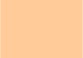 F, factor*.

Table 4 illustrates the clear overlap found between AQ and SPQ subscales, particularly in Factor 1. AQ subscales (communication, social skills, and attention switching) and all SPQ subscales but odd beliefs (cognitive–perceptual) loaded on Factor 1. Factor 1 will be referred to as *Social Disorganization*. Factor 2 comprised cognitive-perceptual subscales odd beliefs, unusual perceptual experiences, and ideas of reference, as well as attention to detail from the AQ, with weak contributions from suspiciousness and odd speech. These factor loadings suggest intrinsic attributes that lead to unusual perceptions, speech and behaviors, and hereafter, Factor 2 will be referred to as *Perceptual Oddities*. Factor 3 was loaded with imagination, attention to detail, and social skills of the AQ, with weaker contributions from constricted affect to social anxiety. Factor 3 will be referred to as *Social Rigidity*. In the subsequent discussions, the structure matrix is referred due to its representation of the relationship between the factors. It is important to note that the ASQ PCA component 2 and component 3 subscales in Table 3 were opposite to ASQ factor analysis Factor 2 and Factor 3 subscales in Table 4.

**Figure 1:**
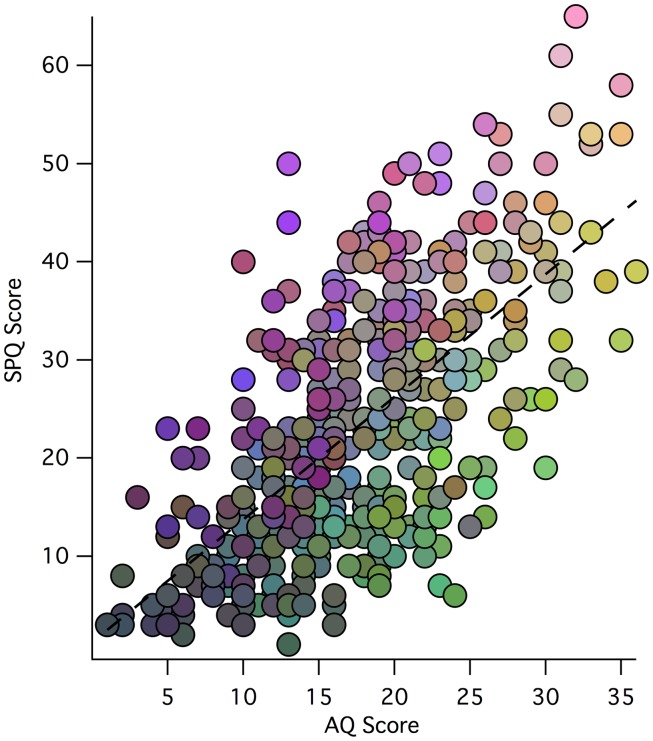
**Scatter plot of participant total SPQ vs. AQ scores: weightings of each participant’s three-factor model scores is indicated by an RGB color model (scaled for each factor), where red represents Factor 1, blue represents Factor 2, and green represents Factor 3 (with the same scaling of factor values to color values for each factor)**. Thus, the low scores in both AQ and SPQ toward the origin tend to be shaded gray, while the extreme AQ and SPQ scores are more illuminant.

The distribution of factor scores across 449 participants can be visualized easily via a RGB color additive model, where red represents Factor 1 (*Social Rigidity*), blue represents Factor 2 (*Perceptual Odditie*s), and green represents Factor 3 (*Social Disorganization*) (see Figure 1). While individual differences in factor scores can be discerned, so can the general correlation between AQ and SPQ scores. Relative to the regression line, diametric tendencies are clearly observed with green/blue shadings to the bottom right and pink/purple/brown shades to the upper left.

Figure 1 is a visual representation of the relationship between SPQ and AQ scores for the 449 participants. The overall correlation between scores is evident through the main trend of the data points. The colors in the plot represent how each participant scored on the three factors of the Factor Analysis. It is clear that those with more *Social Rigidity* scored higher on total AQ and lower on total SPQ. Similarly, those with more *Perceptual Odditie*s had higher total SPQ and lower total AQ. Shared *Social Disorganization* is seen along the line of best fit and into higher SPQ reflecting a relationship between the factors, and supporting the overall relationship between autism and schizophrenia spectrum disorders.

### Comparing the PCA structures of the ASQ and ASBQ

To directly compare with Dinsdale et al. (35), we replicated the AQ and SPQ-BR subscales (ASBQ) and ran a PCA. A three-component solution was revealed, similar to our full-scale ASQ PCA, which explained 56.15% of the variance. However, there were some differences between the models (see Table S1 in Supplementary Material). The most significant change in the component structure from the ASQ to the ASBQ was the transfer of subscales no close friends and constricted affect from the ASQ component 1 to ASBQ component 2. In restricting these two subscales to one no close friends/constricted affect subscale, it fell in line with AQ subscales attention to detail, imagination, and social skills. Furthermore, social skills contributed more to component 2 than component 1 in the ASBQ compared to the ASQ PCA.

### Questionnaire and factor correlations

Pearson correlations between participant scores on the three factors (*Social Desirability, Perceptual Oddities, and Social Rigidity*), total AQ, total SPQ, and the SPQ dimensions are shown in Table 5.

**Table 5:** **AQ and SPQ correlations with factor analysis factors**.

	Social desirability	Perceptual oddities	Social rigidity
Total AQ	0.75**	0.15**	0.76**
Total SPQ	0.96**	0.51**	0.30**
Interpersonal	0.75**	0.08	0.42**
Cognitive–perceptual	0.66**	0.64**	0.08
Disorganized	0.62**	0.16*	0.07
Social desirability	–	0.3*	0.35*
Perceptual oddities	0.3*	–	−0.03
Social rigidity	0.35*	−0.03	–

*p < 0.05, **p < 0.001.

Strong correlations were evident between *Social Disorganization*, and AQ and all SPQ dimensions. There were strong correlations between *Perceptual Oddities*, and SPQ total and cognitive–perceptual subscales, this relationship was weak for total AQ. Finally, there was a strong correlation between *Social Rigidity* and total AQ, but weak for total SPQ. There was a weak positive relationship between *Social Disorganization*, and *Perceptual Oddities* and *Social Rigidity*, with no relationship present between *Perceptual Oddities* and *Social Rigidity*.

## Discussion

This study was the first to investigate the contributions of autistic and schizotypal traits through a combined, randomized autism schizotypal questionnaire (ASQ). We revealed a robust three-factor solution, as opposed to Dinsdale et al.’s (35) two-components, in the analysis of only shared variance between the subscales. The correlation and factor analyses provided face value support for a shared fundamental phenotype in autism and schizophrenia spectrum disorders; *Social Disorganization* (3, 5, 34, 35). Further, they exposed independent positive schizotypy and autistic rigidity phenotypes. This evidence brings to light an important question: does the relationship between autistic and schizotypal scores result from a common phenotype, or is it due simply to a lack of differentiation between distinct symptoms by measurement tools?

As expected, the first factor, *Social Disorganization*, supported a comorbid social–cognitive dysfunction central to autism and schizophrenia spectrum disorders (5, 16–19, 35). *Social Disorganization* included social AQ and all SPQ subscales but odd beliefs, explaining the majority of the variation in the subscale scores. The autism and schizophrenia spectra are presented in current diagnostic tools as completely separate disorders. Therefore, diagnosis relies on the subjective interpretation of symptoms that are specific to autism and schizophrenia spectrum disorders, excluding the shared phenotype. Clearly, relying on subjective symptom assessment risks confusion and misinterpretation, potentially leading to misdiagnosis and mistreatment of symptoms. The trait combination of the *Social Disorganization* factor is reminiscent of schizoid PD, defined by the DSM-IV-TR (12) as exclusively negative schizotypy (prior to its removal from the DSM-5). Core features of schizoid PD are fundamental to the pervasive social dysfunction in autism; lack of interest in, and active avoidance of social situations both in occupation and daily life, restricted affect and empathy, odd communication, and relationship detachment, as well as rigid pursuit of personal interests (12–14). Schizoid PD may be the conceptual or phenotypic link between autism and schizophrenia spectrum disorders, with impairments in empathy, communication oddities, social isolation, and mental rigidity some of the common central features (13, 14). Social anhedonia is another core feature of schizoid PD (24, 25, 73), the negative aspects of schizotypal PD (24, 25) and autism (26), which is characterized by atypical interpersonal behaviors. Collins et al. (24) found that social anhedonics have significantly higher scores on schizoid scales than controls; however, social anhedonics do not differ in level of schizotypy. This relationship suggests that the schizoid phenotype provides a more accurate representation of social anhedonia than does schizotypy (24). *Social Disorganization* appears to reflect the schizoid phenotype as a combination of social autistic and Interpersonal schizotypal tendencies. With the addition of disorganization in speech and behavior, *Social Disorganization* links the two spectrum disorders and raises cause for concern over the accuracy of current diagnostic processes.

Disorganized subscales of the SPQ were a substantial contributor to the *Social Disorganization* factor. Disorganization was not a specific criterion for schizoid PD; however, SPQ Disorganized subscales have explained a substantial amount of variance in the AQ, particularly in communication (5, 14) and motor behavior (14). Pervasive interpersonal dysfunction leads to disorganization in speech and behavior, which manifests in the social environment. This indirect effect provides an explanation for the role of disorganized subscales in the first factor. In addition to disorganization, cognitive–perceptual subscales contributed to the first factor, supporting broad shared traits between the disorders (3, 11, 33–35). In both autism and schizophrenia, environmental interpretation plays an integral role in the individual’s experience. Specifically, the misinterpretation of environmental stimuli is evident in both spectra and can be quantified with the SPQ subscale unusual perceptual experience, which has been found to mimic AQ predictors of abnormal sensory responses (4). Furthermore, schizoid individuals report more fantasy and heightened sensitivity experiences (14), explaining its role in the shared *Social Disorganization* factor.

The strength of the relationship between the AQ, and interpersonal and disorganized SPQ subscales, and weak relationship with cognitive–perceptual subscales, further support the underlying schizoid phenotype. These data suggest that the interpersonal and social AQ subscales scores are a reflection of each other, not differential measures of separate traits. Altogether, the correlations demonstrated a clear common *Social Disorganization* that links the two spectra, which can be defined in terms of schizoid PD.

The exclusion of schizoid PD from the DSM 5 was a consequence of little empirical research resulting in only 1% reported pathological prevalence of the disorder (13, 74). Although the diagnosis has been removed, the schizoid phenotype remains a distinct cluster of symptoms (75). The diagnostic exclusion criteria for schizoid PD; independence from schizophrenia, mood disorder with psychotic features, psychotic disorder, and pervasive developmental disorder, may have contributed to its removal from the DSM 5 (12). The very nature of the schizoid phenotype suggests that affected individuals may carry out a life that suits their social preference. Thus, seeking clinical intervention due to increasing symptom severity that is in line with more severe schizophrenia or autism spectrum pathology (13). Although schizoid PD tends to be more stable than schizotypal PD, schizoid symptoms can be prodromal to schizotypal PD, which in turn can be prodromal to more severe schizophrenia spectrum disorders (13, 14). A child presenting with profound negative symptoms (abnormal social interaction and interpersonal skills, lack of eye contact, impoverished language, and restricted range of thought and cognition) may be assigned a diagnosis of autism rather than child-onset schizoid PD, or alternate schizophrenia spectrum disorders. Furthermore, an additional schizophrenia diagnosis to pre-existing autism is possible should odd language and behavior be misinterpreted (47, 48). A diagnosis of autism relies on the individual’s developmental history, and disclosure of this information could be difficult as it depends largely on the mental health of the parent (47). Relying on relatives to provide clinical information can be difficult due to the genetic association between the disorders. Schizoid, paranoid, and schizotypal PDs are more likely in relatives of schizophrenia and ASDs (12, 55). The mental health history of relatives may also lead to the symptoms classification that is in line with genetic predictions. The risk of misdiagnosis due to misinterpretation of social and communication dysfunction is accentuated by the removal of schizoid PD, incorporation of Asperger’s disorder into autism, and removal of stringent age of onset and language development delay criteria in autism (10). These changes increase the variability and ambiguity in differentiating autism and schizophrenia spectrum disorders, thereby increasing diagnostic and therapeutic risks.

The second factor, *Perceptual Oddities*, separated the positive dimension of the schizophrenia spectrum from the shared *Social Disorganization* phenotype. After the relationship between the factors was taken into account, strongest contributions to this factor were from cognitive–perceptual SPQ subscales odd beliefs, unusual perceptions, ideas of reference, and weak suspiciousness. This factor provided some support for Dinsdale et al.’s (35) second component, however, with the absence of autistic subscales in the negative direction their autism-positive schizotypy axis was not supported. Disorganized odd speech loaded weakly also, adding weight to the argument of a differential schizotypal construct. Interestingly, attention to detail had a moderate contribution to *Perceptual Oddities*, as in Dinsdale et al. (35). Cognitive–perceptual features have been found to explain a substantial proportion of the variance in Attention to Detail (5), and those scoring highly may be particularly analytical of details leading to an over-interpretation of reality. Odd beliefs were the strongest contributor to *Perceptual Oddities*, and had no contribution to any other factor. Dinsdale et al. (35) found odd beliefs to be the most significant contributor to their second diametric component, suggesting that this trait may play a key role in the differentiation between autistic and schizotypal tendency. Suspiciousness, however, was not an influential predictor of *Perceptual Oddities*. Instead suspiciousness loaded substantially on the shared factor, *Social Disorganization*. The relationship between suspiciousness and *Social Disorganization* may be explained by the continual social distress, insecurities, and anxiety that lead to increased suspiciousness in children with autism, which remain to adulthood (4). The strength of the relationship between *Perceptual Oddities* and cognitive–perceptual subscales, but not total AQ, interpersonal and disorganized subscales, suggested this phenotype was specific to psychosis.

Together, the AQ subscales imagination, attention to detail, and social skills made up the third factor, *Social Rigidity*, which was exclusively autistic until the correlation between factors was taken into account. The factor correlations revealed a contribution, although weak, from all interpersonal subscales. *Social Rigidity* reflected the rigidity of thought, restricted, and repetitive behaviors, and social dysfunction that are key criteria for ASDs. This phenotypic construct was not found in Dinsdale et al.’s (35) restricted analysis. The AQ subscale imagination was only a moderate contributor to the *Social Rigidity* factor and had weak correlations across all subscales. This finding opposed the diametric model for rigidity of thought in autism and fluidity of thought in schizophrenia, as imagination was not diametric to AQ subscales (2, 5). Individuals with schizophrenia, as well as those with autism, report higher rigidity of thought as measured by imagination than controls (3, 32). This is perhaps a result of a deficit in the active control of imaginative thought in schizophrenia, while representing a lack of diversity in imagination in autism (3). Wolff et al. (14) reported high levels of fantasy in schizoid participants, but also rigidity of mental set, symptoms that are seen in schizophrenia and autism, respectively. Altogether, these imagination traits were highly reported across spectrum groups, but tap into differential thought processes, questions the specificity of the imagination subscale (32). Dinsdale et al.’s (35) data did not produce this autism-specific component, nor did their analysis of Wakabayashi et al.’s data. Instead, the autism-specific subscales loaded negatively against positive schizotypy subscales in a diametric second component, implications of which will be discussed below. *Social Rigidity* had a strong relationship with total AQ, but a weak relationship with total SPQ and its three dimensions, thus represented more classically autistic features.

Our restricted ASBQ PCA, conducted to contrast with Dinsdale et al. (35) and factor analysis as a data reduction technique, revealed some observable differences to the ASQ PCA. First, our PCA’s second and third components are in reverse to those of our factor analysis. Second, the combined and restricted constricted affect/no close friends subscale shifted from component 1 in the ASQ PCA, to component 2 in the ASBQ PCA. This shift renders the subscale more “autistic” and thereby reduces its distinction between autistic tendency and negative schizotypy. Third, the ASBQ third component was almost exclusively loaded with odd beliefs, suggesting that odd beliefs are a separate phenotype of schizotypy in the restricted model. Odd beliefs are culturally and sample sensitive (5, 12), thus it is important to specify that these data were taken from an Australian population, while Dinsdale et al. (35) took their sample from Canadian Undergraduate students. Fourth, The AQ subscale attention to detail was strongly loaded on the second component for both ASQ and ASBQ models as a diagnostically specific autistic trait. However, Dinsdale et al. (35) suggested that attention to detail represents an independent dimension of autism, as the subscale did not contribute to either component in their restricted model. Finally, the ASBQ subscales explained less of the model variance than did the ASQ. This was particularly true of Component 1, providing further support that the full-scale questionnaire is a more comprehensive assessment of autistic and schizotypal traits. Due to the differences in dimension reduction process between PCA (using unique plus shared variance) and factor analysis (only shared variance), the factor analysis subscales explained slightly less of the total variation than the subscales in PCA extraction (42, 43, 45). While PCA is a suitable tool for analyzing datasets without *a priori* assumptions about the existence of underlying constructs, we argue that the use of factor analysis here was a superior method for this type of dataset, as it exposes underlying constructs in autistic and schizotypal tendency (42, 43, 45). Thus, the three-factor model was clearly a more accurate representation of shared and differential traits.

Dinsdale et al. (35) identified a shared social–communication disinterest, impairment, and abnormality despite their use of the SPQ-BR and PCA technique. This indicates that the common *Social Disorganization* phenotype is robust across instruments. However, we argue that the full-scale ASQ factor structure provides a more comprehensive representation of autistic and schizotypal tendency, as it more accurately reflects the underlying constructs that characterize the two spectra. Furthermore, the *Perceptual Oddities* and *Social Rigidity* phenotypes were somewhat unrelated, rather than diametric. Thus, these findings provide evidence against the diametric model of autism and schizophrenia (3, 5, 33, 34) and Dinsdale et al.’s (35) diametric autism-positive schizotypy axis. The underlying constructs identified in this study supported literature reporting positive schizophrenia symptoms in autism, and autistic symptoms in schizophrenia (4, 6, 11, 33, 34). The ASQ has shown a clear separation of disorder specific traits, thus may be a useful tool for distinguishing autistic and schizotypal tendency that could be validated in the clinical setting. However, the ASBQ also extracted three factors, suggesting that it is not merely the use of the full-scale instrument that exposes the differential factors.

The inclusion of a “neutral” response option in the SPQ-BR presented by Dinsdale et al. (35) creates noise in the data that may have resulted in their diametric second component. Forcing an affirmative or negative response, as in our 4-point scale, provided better discriminant value than a scale with a “neutral” response. Furthermore, it would expose those that tend to respond in a socially desirable manner despite possessing certain trait. Wakabayashi et al. (34) presented their questionnaires separately in their original form, with the SPQ in a “yes/no” forced choice format. With only two response options and the absence of reverse scored items in the SPQ, a bias to a socially desirable “no” response is possible. Moreover, a Likert scale design has been shown to improve internal reliability and convergence of the SPQ (70). In combining the AQ and SPQ, reverse scored items are included and all items are presented on a 4-point Likert scale with the neutral response option removed, thus response bias is reduced. Consequently, these data better represented the relationship between autistic and schizotypal tendency. The self-report nature of these results provided an individual’s perspective of their own behavior and personal interests, but may be subjected to social desirability bias. The response quality does however reflect a very personal representation to an individual’s thoughts, feelings, and behaviors, perhaps providing a richer response quality than clinically observed behaviors. However, it is possible that retaining 4-point scale in the scores may improve the item-by-item correlations and consequently the reliability and factor analysis (76).

Altogether, with the evident confusion in behavioral overlap between social AQ and interpersonal SPQ at a trait level, which reflects schizoid PD, there is a risk of misdiagnosis in clinical settings. These data highlight the need for care in diagnostic and research settings involving the two spectra, particularly in the recruitment of accurate and distinct sample groups to avoid unbiased conclusions. As imaging research continues to identify neuromarkers specific to social–cognitive function (2, 18, 31), the search for differential neuromarkers to separate social–cognitive dysfunction that distinguish autistic and schizophrenia spectrum disorders is imperative (2, 18, 22). However, in light of the similarity in behavioral phenotypes, researchers must be vigilant to ensure exclusion of possible co-morbidities and misdiagnoses within the participant sample (1, 8, 22). Ultimately, neuromarkers are likely to provide an efficient and effective means for intervention, diagnosis and treatment development (1).

In conclusion, we presented robust evidence for a shared *Social Disorganization* phenotype in autistic and schizotypal tendency that resembles schizoid PD. In addition, we revealed discriminating factors of *Social Rigidity* and *Perceptual Oddities* that represented a specific phenotype in autistic and schizotypal tendency, respectively. This is in contrast to Dinsdale et al.’s (35) diametric component. We suggest that these discriminating factors be validated and applied in neuroimaging studies to identify neuromarkers associated with these factors. The identification of neuromarkers that differentiate autistic and schizotypal traits may ultimately lead to an objective diagnostic tool. This in turn may prevent misdiagnosis arising from the misinterpretation of shared phenotypes.

## Conflict of Interest Statement

The authors declare that the research was conducted in the absence of any commercial or financial relationships that could be construed as a potential conflict of interest.

## Supplementary Material

The Supplementary Material for this article can be found online at http://www.frontiersin.org/Journal/10.3389/fpsyt.2014.00117/abstract
