# Supplementary material for: Factor Analysis Demonstrates a Common Schizoidal Phenotype within Autistic and Schizotypal Tendency: Implications for Neuroscientific Studies
Source: Front Psychiatry. 2014 Aug 27;5:117. doi: 10.3389/fpsyt.2014.00117 (PMC4145657; doi:10.3389/fpsyt.2014.00117)
Supplement: Supplementary file 1 [file Table1.PDF]

1. Supplementary material

Supplementary Table 1: PCA Pattern Matrix of Combined AQ and SPQ-BR Subscales

	Component 1	Component 2	Component 3
Odd Behaviour	.779		
Communication	.767		
Attention Switching	.712		
Ideas of Reference/Suspiciousness	.616		
Odd Speech	.590		
Social Anxiety	.525		
Unusual Perceptual Experience	.488		.452
Attention to Detail		.806	
No Close Friends/Constricted Affect		.655	
Imagination		.648	
Social Skill	.405	.628	
Odd Beliefs			.835
Eigenvalues	4.026	1.553	1.159
% of Variance Explained	33.55%	12.95%	9.66%
Rotation Sum of Square	3.591	2.462	1.427
Total Variance	56.15%		

Subscale: SPQ-BR: (blue) *AQ:* (orange)
